# Effects of a nutritional intervention on impaired behavior and cognitive function in an emphysematous murine model of COPD with endotoxin-induced lung inflammation

**DOI:** 10.3389/fnut.2022.1010989

**Published:** 2022-11-17

**Authors:** Charlotte E. Pelgrim, Ingrid van Ark, Ronja E. van Berkum, Anne M. Schuitemaker-Borneman, Inge Flier, Thea Leusink-Muis, Hamed Janbazacyabar, Mara A. P. Diks, Harry R. Gosker, Marco C. J. M. Kelders, Ramon C. J. Langen, Annemie M. W. J. Schols, Robert J. J. Hageman, Saskia Braber, Johan Garssen, Gert Folkerts, Ardy van Helvoort, Aletta D. Kraneveld

**Affiliations:** ^1^Division of Pharmacology, Faculty of Science, Utrecht Institute for Pharmaceutical Sciences, Utrecht University, Utrecht, Netherlands; ^2^Department of Respiratory Medicine, NUTRIM School of Nutrition and Translational Research in Metabolism, Maastricht University Medical Centre+, Maastricht, Netherlands; ^3^Danone Nutricia Research, Utrecht, Netherlands

**Keywords:** behavior, brain, COPD, nutritional intervention, elastase, inflammation, lung

## Abstract

One cluster of the extrapulmonary manifestations in chronic obstructive pulmonary disease (COPD) is related to the brain, which includes anxiety, depression and cognitive impairment. Brain-related comorbidities are related to worsening of symptoms and increased mortality in COPD patients. In this study, a murine model of COPD was used to examine the effects of emphysema and repetitive pulmonary inflammatory events on systemic inflammatory outcomes and brain function. In addition, the effect of a dietary intervention on brain-related parameters was assessed. Adult male C57Bl/6J mice were exposed to elastase or vehicle intratracheally (i.t.) once a week on three consecutive weeks. Two weeks after the final administration, mice were i.t. exposed to lipopolysaccharide (LPS) or vehicle for three times with a 10 day interval. A dietary intervention enriched with omega-3 PUFAs, prebiotic fibers, tryptophan and vitamin D was administered from the first LPS exposure onward. Behavior and cognitive function, the degree of emphysema and both pulmonary and systemic inflammation as well as blood-brain barrier (BBB) integrity and neuroinflammation in the brain were assessed. A lower score in the cognitive test was observed in elastase-exposed mice. Mice exposed to elastase plus LPS showed less locomotion in the behavior test. The enriched diet seemed to reduce anxiety-like behavior over time and cognitive impairments associated with the presented COPD model, without affecting locomotion. In addition, the enriched diet restored the disbalance in splenic T-helper 1 (Th1) and Th2 cells. There was a trend toward recovering elastase plus LPS-induced decreased expression of occludin in brain microvessels, a measure of BBB integrity, as well as improving expression levels of kynurenine pathway markers in the brain by the enriched diet. The findings of this study demonstrate brain-associated comorbidities – including cognitive and behavioral impairments – in this murine model for COPD. Although no changes in lung parameters were observed, exposure to the specific enriched diet in this model appeared to improve systemic immune disbalance, BBB integrity and derailed kynurenine pathway which may lead to reduction of anxiety-like behavior and improved cognition.

## Introduction

The main characteristics of chronic obstructive pulmonary disease (COPD) include pulmonary inflammation, alveolar wall destruction, and airway remodeling and obstruction (1). In addition, COPD is recognized as a multisystemic disease affecting cardiovascular, musculoskeletal and both cognitive and mental health functions (2–4). One cluster of extra-pulmonary comorbidities of COPD involves the brain, which includes cognitive impairment, depression, and anxiety. These conditions are related to a greater disease progression (5–7), poorer treatment adherence (8–10) and increased mortality (5, 6, 10–13). Therefore, it is of great importance to treat these comorbidities adequately in COPD patients.

In COPD, the disease severity is not exclusively dependent on the aforementioned lung pathologies, but is related to the poor nutritional status of COPD patients as well. Around 25–40% of COPD patients are underweight and 35% have a severely low fat-free mass index (14, 15). The majority of severe COPD patients suffer from nutritional deficiencies, have low lean mass, and are frequently undernourished. Among others this high prevalence of undernutrition is caused by poor dietary quality and lifestyle habits, decreased dietary intake due to anorexia and early satiety, and increased energy expenditure related to circulating factors such as hormones, adipokines, and inflammatory cytokines. Undernutrition and micronutrient deficiencies have been associated with neurodegeneration, and COPD patients with increased nutritional risk are more likely to have cognitive impairment (16).

The occurrence of exacerbations affect disease comorbidities in COPD as well (1). An exacerbation is an acute worsening of disease symptoms and is often induced by an opportunistic bacterial or viral infection (17). Frequently, an exacerbation leads to hospitalization, which further accelerates disease progression (18). The relation between exacerbations and brain-related comorbidities may be bi-directional. On one hand, studies show that brain comorbidities are related to an increased risk for exacerbations and hospitalizations (5, 6, 19, 20). On the other hand, brain comorbidities may be one of the consequences of exacerbations (21–26) and may not restore after recovery, at least not within 3 months (24, 25). Considering that exacerbations have a major impact on the mental health and cognitive function in COPD patients and vice versa, optimal treatment during and after an exacerbation is of great importance to manage both the brain comorbidities and the risk of future exacerbations.

Due to the heterogeneity of the disease, both pharmacological and non-pharmacological treatment options used in COPD patients might not be optimal for all patients (1). Multimodal pulmonary rehabilitation is applied frequently, however, concomitant problems with mental health negatively affect the efficacy and adherence in patients (27–29). In addition, psychotropic drug treatment of mental health problems in COPD specifically is challenging, considering the low efficacy and safety issues (7, 30). Since exacerbations not only enhance pulmonary and systemic inflammation (31, 32), but most likely also neuroinflammation, targeting inflammation in COPD might be pivotal to improve overall health during and after exacerbations.

Systemic inflammation might indeed be one of the driving forces of brain-related problems in COPD (7). The significant involvement of systemic inflammation in brain problems has previously been stressed in disorders like depression and Alzheimer’s disease (33, 34). Several nutritional compounds are known to ameliorate inflammation in diseases like rheumatoid arthritis and inflammatory bowel disease (35, 36). One of these compounds is omega-3 fatty acids, including eicosapentaenoic acid (EPA) and docosahexaenoic acid (DHA), highly concentrated in fish oil. The intake of omega-3 fatty acids is generally low in COPD patients (37–39). In addition, an increased intake of omega-3 fatty acids has been related to lower systemic inflammation (40). These fatty acids can diminish inflammatory processes through acting on inflammatory cells and cytokine production, amongst others in the lungs (36, 41). In addition, omega-3 fatty acids have beneficial effects on processes in the brain, including the reduction of neuroinflammation, improvement of synapse function and support of blood-brain barrier integrity (42, 43). Furthermore, the intake of fiber is low in COPD patients (37, 44, 45), which is related to the severity of decreased lung function (46). Prebiotics can ameliorate inflammatory processes via numerous pathways, in which short-chain fatty acids might play an important role (35). With respect to the behavioral aspects of COPD, the availability of serotonin might be of importance because of its crucial role in a normal mental health function. The precursor of serotonin is the essential amino acid tryptophan and this is provided by the diet. Circulating ratios of kynurenine to tryptophan are elevated in COPD patients, suggesting increased systemic inflammation, and this is associated with disease severity (47). During exacerbations, tryptophan levels are decreased in COPD patients (48). Furthermore, vitamin D levels are low in COPD patients (49, 50), and vitamin D deficiency has been related to disease severity (50) as well as depression (51). This is of importance, because vitamin D does not only play a role in mood regulation (52), but it also has anti-inflammatory and neuroprotective properties (53–55). Considering the side effects and limited efficacy of drugs, especially psychotropic drugs (7), nutritional interventions might provide a useful support in the treatment of COPD and COPD-related comorbidities. Taken together, we hypothesize that a dietary intervention containing omega-3 fatty acids, prebiotics, tryptophan and vitamin D might be beneficial for treatment of systemic inflammation and brain comorbidities in COPD. With this intervention we follow a multitargeted, multinutrient approach that in other disease models appears to be more promising than interventions with individual nutrients (56).

The first aim of this study is to evaluate the impact of emphysema combined with pulmonary inflammatory events on the brain, i.e., behavior, cognition, and neuroinflammation. The second aim is to evaluate the therapeutic potential of a nutritional intervention containing omega-3 PUFAs, prebiotic fibers, tryptophan, and vitamin D to attenuate the degree of (systemic) inflammation and improve brain function and neuroinflammation in the elastase-induced murine model of COPD with lipopolysaccharide (LPS)-induced pulmonary inflammation. Mice were intratracheally exposed to elastase for three times, followed by three intratracheal LPS exposures, to induce emphysema and recurrent pulmonary inflammation, respectively. The open field and T-maze spontaneous alternation tests were performed to assess behavior and cognition, respectively, and post-mortem analyses were executed to evaluate pulmonary, systemic, and neuroinflammation, together with blood-brain barrier integrity.

## Materials and methods

### Animals and experimental design

Male C57Bl/6J mice (*n* = 88; Charles River Laboratories, Germany), 11–13 weeks old were solitarily housed in individually ventilated cages with cage enrichment under controlled conditions with a 12 hours (h) day-night light cycle. All experimental procedures were performed in the light phase. Mice had *ad libitum* access to water and food. All animal procedures were performed in accordance with EU guidelines and national legislation, and approved by the Central Committee for Animal Experiments (CCD) and Animal Welfare Body (IVD) of the Utrecht University, Netherlands (AVD1080020184785).

**FIGURE 1:**
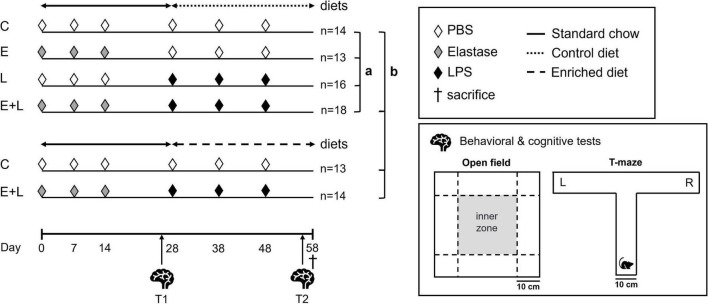
Experimental design. Mice were intratracheally (i.t.) instilled with elastase or vehicle on days 0, 7, and 14. Mice received LPS or vehicle i.t. for three times on days 28, 38, and 48. The nutritional intervention was applied from the first LPS trigger onward until sacrifice at day 58. The following treatment groups were included: control (C), elastase control (E), LPS control (L), and elastase plus LPS (E + L). Additional C and E + L treated mice were exposed to a nutritional intervention. From day 0 until day 57, mice were fed a standard chow diet (solid line). The enriched diet (rough dashed line) or an isocaloric, isonitrogenous control diet (fine dashed line) was applied from the first LPS trigger onward until sacrifice at day 58. Behavioral and cognitive tests were performed at two time windows: just before the first LPS trigger (T1; days 25–27) and at the end of the study (T2; days 55–57). The open field and T-maze spontaneous alternation tests were used to assess novelty-induced behavior and cognition, respectively. Statistical analyses were performed in two steps, according to the two goals of this study: (a) to assess the impact of the two factors elastase and LPS used to induce COPD, (b) to assess the efficacy of a dietary intervention to improve lung, systemic and brain outcomes in this COPD model.

Animals were randomly divided into six treatment groups (Figure 1). Mice received three weekly doses of 3 U porcine pancreatic elastase (Elastin Products Company, Inc., Owensville, MO, USA) dissolved in 50 μl sterile phosphate-buffered saline (PBS) or solely PBS (Figure 1: experimental day 0, 7, and 14) via intubation-mediated intratracheal (i.t.) instillations, as previously described (57). Two weeks after the last instillation, 2 μg lipopolysaccharide (LPS, from Escherichia coli serotype O55:B5; Sigma-Aldrich, St. Louis, MO, USA) per gram mouse dissolved in 50 μl sterile PBS or solely PBS was administered via i.t. instillations for three times with a 10-day interval (day 28, 38, and 48). Two treatment groups, i.e., control and elastase plus LPS treated mice (E + L), were exposed to the nutritional intervention from the first LPS instillation onward until sacrifice at day 58, 10 days after the third and last LPS instillation. The other four treatment groups were concomitantly fed a isocaloric and isonitrogenous control diet. Behavioral and cognitive tests were performed during two time windows: 11–13 days after the last elastase instillation (T1: day 25–27) and 7–10 days after the last LPS instillation, at the end of the study (T2: day 55–57).

### Dietary intervention

From day 0 until 27, all mice were fed standard AIN-93M pellets (SSNIFF, Soest, Germany). From day 28 onward, an AIN based diet enriched with omega-3 fatty acids, prebiotic fibers, tryptophan and vitamin D (enriched diet, for detailed composition see Supplementary Table 1) was applied in additional C and E + L treatment groups. Mice in the other four treatment groups, i.e., C, E, L, and E + L, were fed an isonitrogenous and isocaloric control diet simultaneously (Figure 1). Food was refreshed once a week.

### Open field test

The open field test was performed on days 25 and 55 as previously described (58). Tests were performed in the light phase during the first half of the day and the order of testing was standardized for T1 and T2. After placement in the center, mice were allowed to explore a gray open field (45 cm × 45 cm) for 5 min. The open field was cleaned with 70% ethanol after every run. Video tracking software (EthoVision XT 14.0, Noldus, Wageningen, Netherlands) was used to analyze the recordings. An inner zone in the middle of the open field of 25 cm × 25 cm was established digitally (Figure 1). By post-acquisition analysis, parameters including total distance moved, and frequency of entering and time spent in the inner zone and corners were determined.

### T-maze spontaneous alternation test

To assess cognitive function, the T-maze spontaneous alternation test was performed on days 26–27 and 56–57, as described previously (59). Tests were performed in the light phase during the first half of the day and the order of testing was standardized for T1 and T2. The T-maze arena consisted of one start arm (49 cm long × 10 cm wide × 20 cm high) and two lateral arms (30 cm long × 10 cm wide × 20 cm high). The mouse was placed in the beginning of the start arm, facing the 10 cm wide wall. After that, the mouse was able to choose between the left or right goal arm (Figure 1). When the mouse had entered one goal arm, the mouse was confined for 30 s in the goal arm by lowering the door. The mouse was then returned to the home cage and the T-maze was cleaned with 70% ethanol. After 2 min, the mouse was placed back in the start arm and was again able to choose one of the goal arms. Successful alternation was considered when the mouse had alternated in the second run compared to the first run within one trial. A total of six trials – three trials per day on two consecutive days – were performed, and the alternation ratio was calculated by dividing the number of alternated trials by the total number of trials.

### Bronchoalveolar lavage

Mice were roughly divided into two groups because of the different tissue processing techniques for the lungs; one group was used for bronchoalveolar lavage (BAL) harvest, and one group was used for emphysema assessment (described in “Assessment of emphysema”). After anesthesia with ketamine (196.8 mg/kg; Narketan, Vetoquinol S.A., Magny-Vernois, France) and medetomidine (1.32 mg/kg; Dexdomitor, Vetoquinol S.A., Magny-Vernois, France) mixture, mice (*n* = 43) were sacrificed by maximal blood collection from cardiac puncture. The trachea was cannulated and lungs were lavaged with 1 ml 0.9% NaCl (37°C) supplemented with a protease inhibitor cocktail (Complete Mini, Roche Diagnostics, Germany) for cytokine measurements. Afterward, lungs were lavaged three times with 1 ml 0.9% NaCl (37°C). All lavages were centrifuged (400 × g, 5 min), and pellets were pooled within the same animal and resuspended in saline to isolate BAL cells. Supernatant from the first ml was stored in −80°C until subsequent cytokine measurements. To count the total BAL cell numbers, a Bürker-Türker chamber was used. Subsequently, cytospins were prepared and stained with DiffQuick™ (Merz and Dade A.G., Switzerland) to determine the number of specific types of BAL cells. An observer differentiated the cells into macrophages, neutrophils and lymphocytes in each sample using standard morphology. Approximately 200 cells were counted and the absolute number for each cell type was calculated based on the totally lavaged BAL cell counts of the corresponding sample (60). The following post-mortem analyses were performed in this subset of mice: cytokine/chemokine levels in serum and BALF, T cell populations in spleen, and tight junction protein expression levels in brain microvessels.

### Assessment of emphysema

After anesthesia with ketamine (196.8 mg/kg; Narketan, Vetoquinol S.A., Magny-Vernois, France) and medetomidine (1.32 mg/kg; Dexdomitor, Vetoquinol S.A., Magny-Vernois, France) mixture, mice (*n* = 45) were sacrificed by maximal blood collection from cardiac puncture. Using a cannula, lungs were fixed with 10% formalin at a constant pressure of 25 cm H_2_O for at least 5 mins. Subsequently, left lobes of the lungs were stored in 10% formalin for a minimum of 24 h. After paraffin embedding, sections of 5 μm were cut and stained with hematoxylin/eosin. The average inter-alveolar distance was determined in six random photomicroscopic images per sample using a reference grid as previously described (60). The grid length was divided by the number of alveolar wall-grid line intersections to calculate the mean linear intercept (Lm). The following post-mortem analyses were performed in this subset of mice: cytokine/chemokine levels in serum, T cell populations in spleen, microglia activation in the brain, and mRNA levels in the brain.

### Cytokines and chemokines in serum and bronchoalveolar lavage fluid

Keratinocyte chemoattractant (KC), tumor necrosis factor alpha (TNF-α), and vascular endothelial growth factor A (VEGF-A) levels were determined in both serum and BALF using a mouse multiplex assay according to the manufacturer’s instructions (ProcartaPlex, Thermo Fisher Scientific, Vienna, Austria). Furthermore, C-reactive protein (CRP) levels were measured in both BALF and serum using an enzyme-linked immunosorbent assay according to the manufacturer’s instructions (R&D Systems, Minneapolis, MN, USA).

### Isolation and flow cytometry of immune cells

Spleens of all animals were isolated and crushed through a 70 μm nylon strainer using a syringe. The resulting cell suspensions were incubated with lysis buffer (eBioscience, Thermo Fisher Scientific, San Diego, CA, USA) for 5 min on ice to remove red blood cells. Cells were washed and resuspended in RPMI 1640 (Lonza, Basel, Switzerland) supplemented with 10% heat-inactivated fetal bovine serum (FBS). Cells were counted and cell suspensions of 10^7^ cells/ml were prepared. After washing with PBS, cells were incubated with CD16/CD32 (Mouse BD Fc Block, BD Biosciences, San Jose, CA, USA) in PBS with 1% bovine serum albumin and 5% FBS to block non-specific binding. Cells were then extracellularly stained with CD4-BV510 (BD Biosciences), CD69-PE-Cy7, CXCR3-PE (both from eBioscience), T1ST2-FITC (MD Bioproducts, St. Paul, MN, USA) and CD196-PE (Biolegend, San Diego, CA, USA). For intracellular staining, cells were fixed and permeabilized with Foxp3 staining buffer set (eBioscience) according to the manufacturer’s instructions. This was followed by incubation with RorγT-Alexa Fluor 647 (BD Biosciences). Using the fixable viability dye eFluor^®^ 780 (eBioscience), viable cells were stained and dead cells excluded. Results were obtained using the FACS Canto II flow cytometer (BD Biosciences) and analyzed using FlowLogic software (Inivai Technologies, Mentone, VIC, Australia). The gating strategy for selecting cell populations is presented in Supplementary Figure 1.

### Microvessel isolation

After sacrifice, brains of mice used for BAL harvest were isolated, snap frozen using isopentane, and stored at −80°C. The brain microvessel isolation is adapted from previously published protocols (61–63). Meninges and cerebella were removed and brains (*n* = 36) were homogenized in ice-cold Dulbecco’s Modified Eagle Medium (DMEM; Gibco; Thermo Fisher Scientific, Waltham, MA, USA) using a glass tissue grinder with small clearance pistil (KIMBLE^®^, Wertheim, Germany) for 30 up-and-down strokes. Subsequently, 5 ml DMEM was added, and samples were centrifuged for 20 min (4,300 × g, 4°C). The pellet was resuspended in 7 ml 18% dextran (MW 100,000; Sigma-Aldrich, St. Louis, MA, USA) in DMEM, and 5 ml DMEM with 31% dextran was added on top. The samples were again centrifuged for 20 min at 4,300 × g (4°C, brake off) in order to create a dextran gradient. The interphase and lower phase were collected and filtered through a 70 μm filter covered with a layer of glass beads (450–600 nm, Sigma-Aldrich, USA). Beads were collected in PBS and centrifuged for 20 min (4,300 × g). After resuspension in 0.5 ml PBS, the aqueous phase was collected and centrifuged for 10 min at 14,000 rpm. The resulting pellet of microvessels was resuspended in 100 μl lysis buffer (15% protease inhibitor solution in RIPA buffer) and homogenized using a tissue homogenizer (Precellys 24, Bertin Instruments, France).

### Western blot

Protein concentrations of homogenized microvessel supernatants were determined using a Pierce BCA protein assay kit (Thermo Fisher Scientific, Waltham, MA, USA). Equal amounts of protein were loaded into a midi Criterion precast protein gel (4–20%, Bio-Rad, Hercules, CA, USA), separated by gel electrophoresis and subsequently transferred onto a polyvinylidene difluoride membrane (Bio-Rad, Hercules, CA, USA) using the Trans-Blot Turbo system (Bio-Rad, Hercules, CA, USA). At room temperature, blots were blocked with 5% milk powder in PBS containing 0.1% Tween 20 for at least 1 h. Next, blots were incubated with primary antibodies for β-actin (1:2,000; #4970, Cell Signaling Technology, Danvers, MA, USA), claudin-5 (1:500; #34-1600, Invitrogen, Waltham, MA, USA), occludin (1:500; #40-4700, Invitrogen, Waltham, MA, USA) or zonula occludens-1 (ZO-1) (1:750; #40-2200, Invitrogen, Waltham, MA, USA). For detection, horseradish peroxidase conjugated secondary antibodies and enhanced chemiluminescence substrate were applied. Quantification of band intensity was performed using ImageJ 1.52p. For each protein, peak ratios to β-actin were calculated to compare relative expression levels.

### Immunohistochemistry and image analysis in brain

Brains of the mice used for emphysema assessment were isolated after sacrifice. Left hemispheres were snap frozen using isopentane and stored at −80°C until further processing. Right hemispheres were fixed in 10% formalin for at least 24 h followed by 30% sucrose in PBS until sectioning using a cryostat. Forty μm thick coronal sections were incubated overnight with blocking serum followed by 24 h incubation with rabbit anti-iba-1 (1:1,000; #019-19741, Wako Chemicals, Neuss, Germany). Subsequently, sections were incubated for 2 h with fluorophore tagged donkey anti-rabbit secondary antibody (1:500; #A-31573, Invitrogen, Waltham, MA, USA). Prolong Gold mounting medium containing DAPI (Invitrogen, Waltham, MA, USA) was used to embed the sections.

In the amygdala, cingulate cortex – the equivalent of the anterior cingulate cortex (ACC) and part of the prefrontal cortex (PFC) – and CA1 area of the hippocampus, microglial cells were captured by imaging z-stacks with a step size of 1 μm using a confocal microscope (Leica TCS SP8 X) (64). Approximately 4 z-stacks per brain area were acquired in two sections. At least 12 cells per brain area were included and only microglial cells that were fully included within the z-stack were analyzed. Using the semi-automated analysis with the MATLAB script 3DMorph (65), microglial cell volume and ramification index – a measure of activation – were assessed in a blinded manner. In addition, the number of all the cells fully included in the stack were determined per surface area to assess the occupancy.

### RNA isolation and PCR analysis

Prefrontal cortices from left hemispheres (*n* = 6–7 per group) of animals used for emphysema assessment were isolated and homogenized using a tissue homogenizer (Precellys 24, Bertin Instruments, France). RNA was isolated using the RNeasy mini kit according to manufacturer’s instructions (Qiagen, Hilden, Germany). RNA was reverse transcribed with the Tetro cDNA synthesis kit (GC Biotech) by using oligo-dT priming, 400 ng RNA input and incubating 30 mins at 45°C. cDNA was diluted 1/50 in nuclease free water. 4.4 μl of cDNA dilution was loaded into 384 well plates (Hardshell plates, Biorad). Primers (for sequences see Supplementary Table 2) were added with Sensimix qPCR mastermix with SyBr-green (GC Biotech). Plates were run on a LightCycler 480 (Roche) with the following cycling conditions: 10′95°C (initial denaturation), 45 × 10″95°C, 20″60°C, followed by a melting curve determination. Melting curves were analyzed with the LightCycler480 software. The efficiency-corrected target quantity (N0) values were determined with the LinRegPCR program. The expression of genes of interest was normalized by geometric averaging of reference genes (*Cyclo, RPLP0, B2M*, and *HPRT*) by GeNorm software. Relative expression levels to the control group were finally calculated.

### Statistical analysis

For differences between PBS and elastase-exposed mice for the open field and T-maze spontaneous alternation test parameters at T1, independent samples *t*-tests were used. For all endpoint parameters at T2 or post-mortem, analyses were performed in two steps, according to the two goals of this study: to assess the impact of the two factors elastase and LPS used to induce the COPD model, and to assess the efficacy of a dietary intervention to improve lung, systemic and brain outcomes in this COPD model. For effects of elastase and/or LPS exposures, a first two-way ANOVA was used with groups C, L, E, and E + L (indicated with “a”). For diet effects, a second two-way ANOVA was performed with both model and diet as factors including groups C, C + enriched diet, E + L and E + L + enriched diet (indicated with “b”). Bonferroni corrections were applied to correct for multiple comparisons. When the assumption for normality was violated, an appropriate transformation or the non-parametric Mann-Whitney *U* or Kruskal-Wallis test with Dunn’s correction was applied. All analyses were performed using Graphpad Prism version 9. Data are expressed as mean ± SEM and significance was considered when *p* < 0.05.

## Results

### Emphysema

**FIGURE 2:**
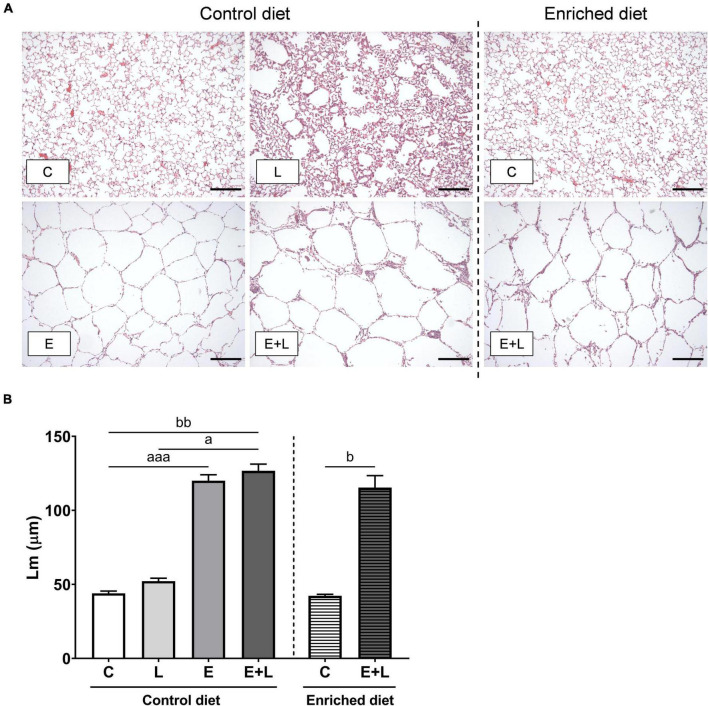
Emphysema assessment in lung histological sections. **(A)** Representative photomicrographs of H&E-stained lung sections for each study group (scale bar: 100 μm; 100× magnification). **(B)** Lm analysis on H&E-stained lung sections. Data are presented as mean (μm) ± SEM. *^a^p* < 0.05; *^aaa^p* < 0.001 (first analysis for elastase and/or LPS effects). *^b^p* < 0.05; *^bb^p* < 0.01 (second analysis for diet and/or E + L effects). C = control (control diet: *n* = 7; enriched diet: *n* = 7); L = LPS (*n* = 7); E = elastase (*n* = 8); E + L = elastase plus LPS (control diet: *n* = 9; enriched diet: *n* = 5).

After elastase treatment, the size of the lungs was enlarged (Supplementary Figure 2). To assess emphysema, mean linear intercept (Lm) was measured in histological sections of lung tissue. Photomicrographs of these sections show enlarged airspaces in the elastase-exposed animals (Figure 2A). There was a significant difference between groups (Kruskal-Wallis; *p* < 0.0001), showing that elastase significantly increased Lm (Figure 2B). In addition, the combined elastase plus LPS intervention resulted in an increased Lm when compared to controls, independent of diet (Figure 2B). The LPS exposures did not affect Lm. Intervention with the enriched diet did not affect the elastase plus LPS-induced increased Lm.

### Pulmonary inflammation

**FIGURE 3:**
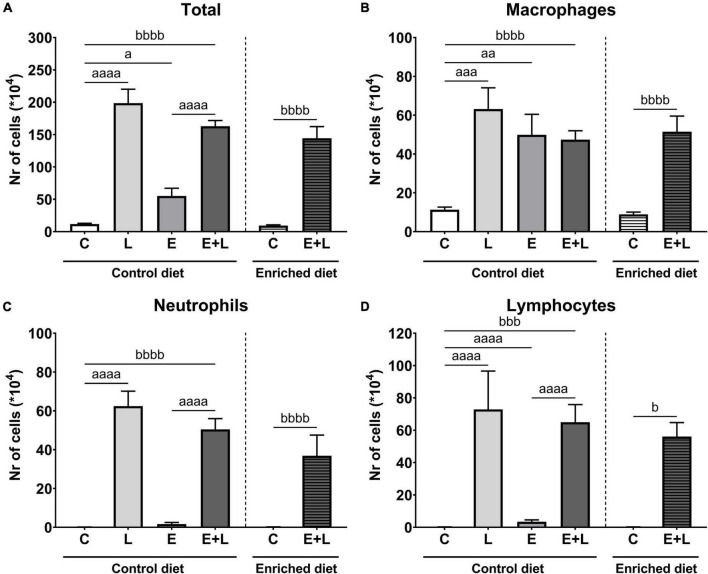
Number of inflammatory cells in BAL at the end of the experiment, 10 days after the last LPS exposure. The total number of cells **(A)** has been determined first. Using this number together with differential cell counts, the number of macrophages **(B)**, neutrophils **(C)**, and lymphocytes **(D)** was determined. *^a^p* < 0.05; *^aa^p* < 0.01; *^aaa^p* < 0.001; *^aaaa^p* < 0.0001 (first analysis for elastase and/or LPS effects). ^ b^*p* < 0.05; *^bbb^p* < 0.001; *^bbbb^p* < 0.0001 (second analysis for diet and/or E + L effects). Data are presented as mean ± SEM. C = control (control diet: *n* = 7; enriched diet: *n* = 6); L = LPS (*n* = 6); E = elastase (*n* = 8); E + L = elastase plus LPS (control diet: *n* = 9; enriched diet: *n* = 7).

To assess the degree of inflammation in the lungs, the number of inflammatory cells were quantified in the BAL. Based on the total number and differential cell counting, the number of specific types of inflammatory cells was determined. LPS exposure had a significant effect on the total number of cells in BAL (*p* < 0.0001). In addition, there was a significant elastase*LPS interaction effect (*p* < 0.01). Multiple comparisons revealed significantly higher total cell numbers in all treatment groups when compared to controls and was highest when mice were exposed to LPS (Figure 3A).

For macrophages and neutrophils, there was a significant effect of LPS (*p* < 0.01 and *p* < 0.0001, respectively), including an elastase*LPS interaction effect (*p* < 0.01 and *p* < 0.05, respectively). A significantly higher number of macrophages was observed in solely elastase- or LPS-exposed mice compared to PBS controls (Figure 3B). Multiple comparisons revealed a significantly higher neutrophil number in both LPS- and elastase plus LPS-exposed mice compared to the respective control group (Figure 3C). Finally, significant effects of both elastase and LPS (*p* < 0.0001) and an elastase*LPS interaction effect (*p* < 0.0001) were observed for lymphocyte numbers, showing significantly higher numbers of lymphocytes in either elastase- or LPS-exposed mice but also mice exposed to both elastase plus LPS compared to the respective control (Figure 3D).

The model has a significant effect on total, macrophage and neutrophil numbers (*p* < 0.0001), and this model effect was present in both the control and enriched diet exposed animals (Figures 3A–C). Significant differences in lymphocyte numbers between groups were observed (Kruskal-Wallis; *p* = 0.0001) and multiple comparisons showed a significantly higher number of lymphocytes in elastase plus LPS-exposed mice compared to controls independent of the diet (Figure 3D). Overall, no effects of the enriched diet were observed for any of the inflammatory cell types.

**FIGURE 4:**
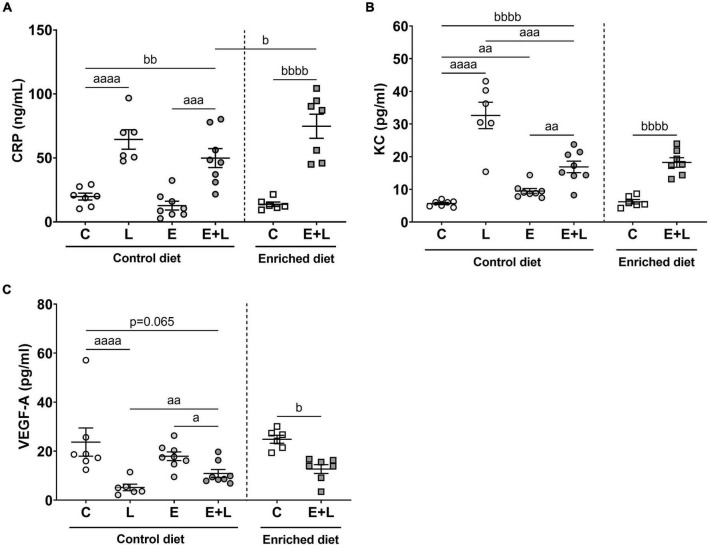
Levels of inflammatory markers in BALF at the end of the experiment, 10 days after the last LPS exposure. The levels of CRP **(A)**, KC **(B)**, and VEGF-A **(C)** are presented. Data are presented as mean ± SEM. *^a^p* < 0.05; *^aa^p* < 0.01; *^aaa^p* < 0.001; *^aaaa^p* < 0.0001 (first analysis for elastase and/or LPS effects). *^b^p* < 0.05; *^bb^p* < 0.01; *^bbbb^p* < 0.0001 (second analysis for diet and/or E + L effects). C = control (control diet: *n* = 7; enriched diet: *n* = 6); L = LPS (*n* = 6); E = elastase (*n* = 8); E + L = elastase plus LPS (control diet: *n* = 8; enriched diet: *n* = 7).

As a second assessment of pulmonary inflammation, levels of the inflammatory mediators CRP, KC, TNF-α, and VEGF-A were measured in BALF. For TNF-α, most samples were below the detection limit (data not shown). There was a trend in an overall effect of elastase (*p* = 0.069; *p* = 0.065) and a significant main effect of LPS (*p* < 0.0001) on CRP and VEGF-A levels in BALF. In addition, a significant interaction effect between elastase and LPS was observed for VEGF-A (*p* < 0.01). A significant effect of LPS and interaction effect between elastase and LPS on KC levels were found (*p* < 0.0001). For both CRP and KC, significantly higher levels were observed in LPS- and elastase plus LPS-exposed mice as compared to the respective control (Figures 4A,B), whereas in mice exposed to elastase plus LPS lower KC levels were observed compared to LPS-exposed mice (Figure 4B). In addition, elastase-exposed mice showed higher KC levels as opposed to controls (Figure 4B). Lower levels of VEGF-A in LPS-exposed and elastase plus LPS-exposed mice as compared to the respective controls were observed (Figure 4C). Though, this effect was less pronounced in elastase plus LPS-exposed mice, since these mice showed higher VEGF-A levels as compared to LPS-exposed mice (Figure 4C).

The model had a significant effect on all inflammatory mediators (*p* ≤ 0.0001). For CRP and KC, increased levels, and for VEGF-A reduced levels, were observed in elastase plus LPS-exposed mice as compared to PBS-exposed controls (Figure 4). There was a significant interaction effect of the model and diet on CRP levels (*p* < 0.05), showing higher levels of CRP in elastase plus LPS-exposed mice fed the enriched diet as compared to animals fed the control diet (Figure 4A). For KC and VEGF-A, no effects of the enriched diet were observed.

### Systemic inflammation

**FIGURE 5:**
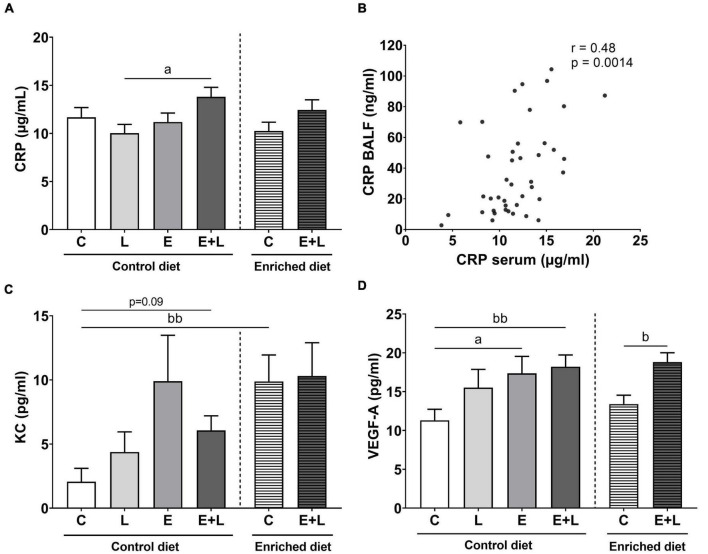
Cytokine levels in serum at the end of the experiment, 10 days after the last LPS exposure. The levels of CRP **(A)**, KC **(C)**, and VEGF-A **(D)** have been determined. A significant correlation between CRP levels in BALF and in serum was observed **(B)**. Data are presented as mean ± SEM. *^a^p* < 0.05 (first analysis for elastase and/or LPS effects). *^b^p* < 0.05; *^bb^p* < 0.01 (second analysis for diet and/or E + L effects). C = control (control diet: *n* = 14; enriched diet: *n* = 13); L = LPS (*n* = 13); E = elastase (*n* = 16); E + L = elastase plus LPS (control diet: *n* = 18; enriched diet: *n* = 14).

To examine the presence and degree of systemic inflammation, levels of CRP, KC, TNF-α, and VEGF-A were measured in serum as well. For TNF-α, most samples were below the detection limit (data not shown). A significant elastase × LPS interaction effect on CRP levels was observed (*p* < 0.05). More specifically, higher levels of CRP in elastase plus LPS-exposed mice as compared to LPS-exposed mice were found (Figure 5A). For KC, an overall significant difference between groups was observed (Kruskal-Wallis; *p* < 0.05), although no significant differences in the multiple comparisons test were found (Figure 5C). There was a significant main effect of elastase on VEGF-A levels (*p* < 0.05), showing higher levels of VEGF-A in elastase-exposed mice as compared to control mice (Figure 5D).

Significant overall effects of the model were observed for CRP (*p* < 0.05) and VEGF-A (*p* < 0.0001). Only significantly higher levels of VEGF-A were observed in the serum of elastase plus LPS-exposed mice (Figure 5D), although no differences were observed for CRP (Figure 5A). Diet effects were only observed for KC levels, where control mice fed with the enriched diet showed significantly higher levels (Figure 5C; *p* < 0.01; Kruskal-Wallis). In addition, there was trend toward higher levels of KC in elastase plus LPS-exposed mice compared to control mice fed the control diet (*p* = 0.09; Kruskal-Wallis). Taking all treatment groups together, a significant positive correlation between CRP levels in BALF and CRP levels in serum was observed (Figure 5B; *r* = 0.48; *p* < 0.01). No significant correlations between BALF and serum levels of KC or VEGF-A were observed (data not shown).

### T cell populations in spleen

**FIGURE 6:**
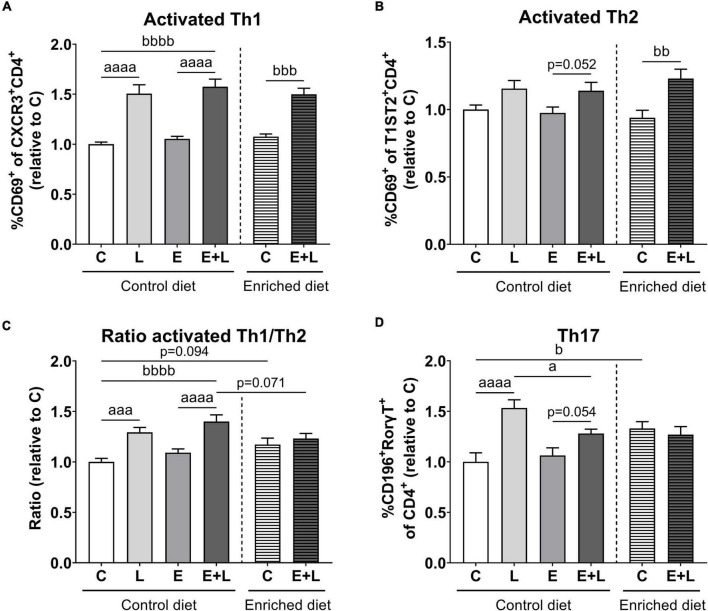
Flow cytometric analysis of T cell populations in the spleen at the end of the study. Relative ratios as opposed to controls of activated Th1 **(A)** and Th2 **(B)** cell percentages, ratios of activated Th1/Th2 cells **(C)** and Th17 cell percentages **(D)** are presented. Data are expressed as mean ± SEM. *^a^p* < 0.05; *^aaa^p* < 0.001; *^aaaa^p* < 0.0001 (first analysis for elastase and/or LPS effects). *^b^p* < 0.05; *^bb^p* < 0.01; *^bbb^p* < 0.001; *^bbbb^p* < 0.0001 (second analysis for diet and/or E + L effects). C = control (control diet: *n* = 14; enriched diet: *n* = 13); L = LPS (*n* = 13); E = elastase (*n* = 16); E + L = elastase plus LPS (control diet: *n* = 18; enriched diet: *n* = 14).

To assess the systemic immune activation, T cell populations in the spleen were measured using flow cytometry. The gating strategy for selecting cell populations is presented in Supplementary Figure 1. There were differences among groups for activated T helper 1 (Th1) cell populations (Kruskal-Wallis; *p* < 0.0001), showing higher percentages of activated Th1 cells in LPS-exposed mice compared to the respective control group (Figure 6A). A higher percentage of activated Th1 cells was observed in mice exposed to elastase plus LPS as compared to controls, independent of diet.

For activated Th2 cells, there was a significant overall effect of LPS (*p* < 0.01) which was mainly present in elastase plus LPS exposed mice as compared to elastase-exposed mice (Figure 6B). In addition, the model had a significant effect (*p* < 0.001) showing an increased percentage of activated Th2 cells in mice fed the enriched diet subjected to elastase plus LPS as compared to PBS controls.

For the ratio of activated Th1/Th2 cells, a significant effect of LPS (*p* < 0.0001) and a trend in an elastase effect (*p* = 0.060) were observed. LPS-exposed mice showed a higher activated Th1/Th2 ratio as compared to the respective control (Figure 6C). In addition, the two-way ANOVA for diet effects revealed a significant model (*p* < 0.001) and model × diet interaction (*p* < 0.01) effect. Elastase plus LPS-exposed mice fed the control diet showed higher activated Th1/Th2 ratios as compared to controls. Furthermore, the enriched diet led to a trend toward lower activated Th1/Th2 ratios in elastase plus LPS-exposed mice compared to control diet fed elastase plus LPS exposed mice (*p* = 0.071; Figure 6C).

A significant overall effect of LPS (*p* < 0.0001) and an elastase × LPS interaction (*p* < 0.05) effect were found for percentages of Th17 cells in the spleen. LPS exposure resulted in increased Th17 percentages in the spleen, although percentages were lower in mice exposed to elastase plus LPS (Figure 6D). In addition, significant differences among the different diet groups were found (Kruskal-Wallis; *p* < 0.05); higher Th17 percentages were observed in control mice fed the enriched diet as opposed to control diet fed mice.

### Open field behavior

To assess exploratory and anxiety-like behavior, the open field test was performed at two time points in the study; at day 25, after elastase exposures (T1), and at day 55, at the end of the study (T2).

At T1, no significant differences in any of the open field parameters were observed in elastase-exposed mice when compared to controls (Supplementary Figure 3).

**FIGURE 7:**
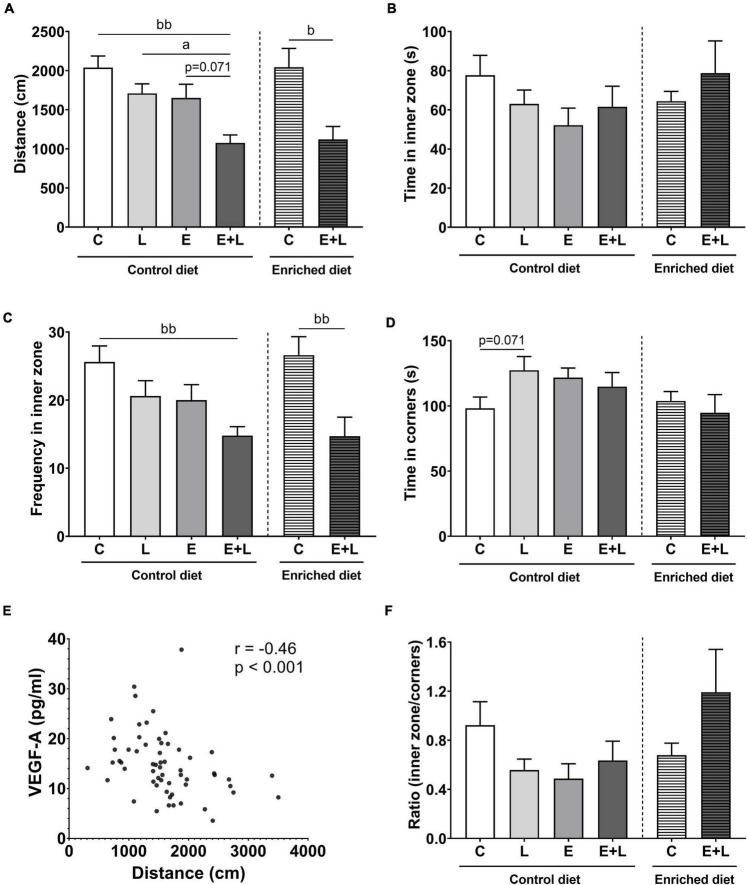
Open field behavior at the end of the study on day 55 (T2). The distance walked **(A)**, time spent in the inner zone **(B)**, frequency of entering the inner zone **(C)**, time spent in the corners **(D)** and the ratio of time spent in the inner zone to time spent in the corners **(F)** of the open field were measured. **(E)** Correlation between distance walked in the open field and VEGF-A levels in serum. Data are expressed as mean ± SEM (*n* = 10 per group). *^a^p* < 0.05 (first analysis for elastase and/or LPS effects). *^b^p* < 0.05; *^bb^p* < 0.01 (second analysis for diet and/or E + L effects). C, control; L, LPS; E, elastase; E + L, elastase plus LPS.

At T2, a trend toward a lower distance walked in elastase plus LPS-exposed mice as compared to elastase- (*p* = 0.071) and LPS- (*p* < 0.05) exposed controls was found (Kruskal-Wallis; Figure 7A). No effects on time spent in the inner zone were observed (Figure 7B). Although for frequency of entering the inner zone a significant overall difference among groups was found (Kruskal-Wallis; *p* < 0.05), no significant differences were observed in the multiple comparisons test (Figure 7C). For time spent in the corners, a trend in the interaction between elastase and LPS was observed (*p* = 0.064), showing a trend toward higher time spent in the corners in LPS-exposed mice as opposed to PBS controls (Figure 7D). The ratio time spent in the inner zone to time spent in corners was calculated to correct for locomotion. No effects of elastase or LPS were observed on this ratio (Figure 7F).

A decrease in the total distance walked was observed in elastase plus LPS-exposed mice compared to control mice independent of diet (Kruskal-Wallis; Figure 7A). An overall effect of the model was observed on the frequency of entering the inner zone of the open field (*p* < 0.0001; Figure 7C), showing lower frequencies in elastase plus LPS-exposed animals compared to control mice. For all parameters, no significant overall effects of the enriched diet were observed (Figures 7A–D). For the ratio time spent in the inner zone to time spent in the corners, a trend toward an interaction effect between the model and enriched diet was observed (*p* = 0.076; Figure 7F). However, no significant differences were found in the multiple comparisons test.

When combining all the groups, a significant negative correlation between distance walked and VEGF-A serum levels was observed (*r* = −0.46, *p* < 0.001; Figure 7E), whereas no significant correlations with the other markers in serum were observed (data not shown).

Comparing results at T2–T1, significant main effects of elastase and LPS separately were found for distance walked and frequency of entering the inner zone (*p* < 0.01 for elastase, *p* < 0.05 for LPS). A trend toward a greater decrease in distance walked in elastase plus LPS-exposed mice compared to solely LPS-exposed mice was observed (*p* = 0.068; Supplementary Figure 4). In addition, a significant overall effect of the model was observed on distance walked and frequency of entering the inner zone (*p* < 0.001 and *p* < 0.01, respectively). A greater decrease in mice exposed to elastase plus LPS compared to control mice fed the control diet was observed (*p* < 0.01 and *p* < 0.05, respectively; Supplementary Figures 4A,C). This effect was not significant in mice fed the enriched diet. No significant effects of elastase or LPS on the ratio time spent in the inner zone to time spent in the corners of the open field were found. Though, in the analysis for diet effects a significant interaction effect between the model and enriched diet was observed (*p* < 0.05; Supplementary Figure 4E). The elastase plus LPS-exposed mice fed the enriched diet showed a higher relative ratio at T2–T1 as compared to mice fed the control diet (Supplementary Figure 4E).

### Cognitive function

**FIGURE 8:**
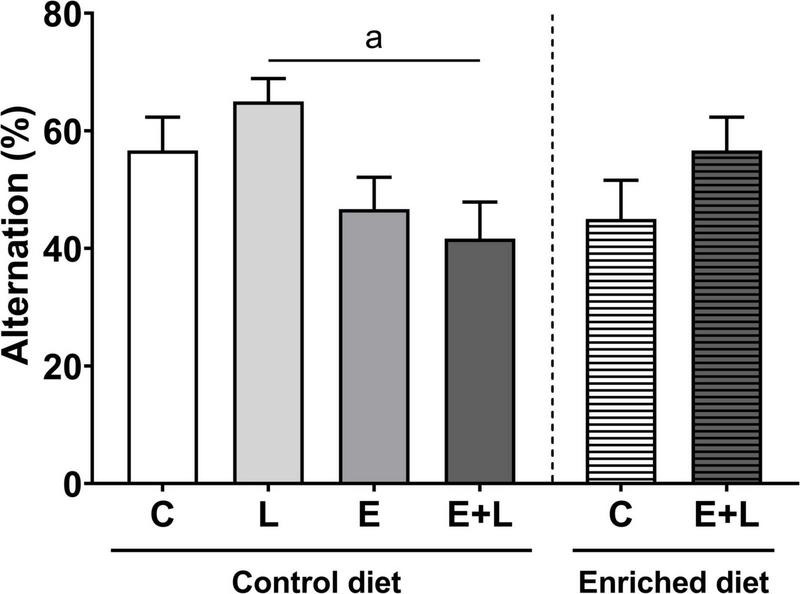
Cognitive function as measured with the T-maze spontaneous alternation test at T2 of the study (days 56–57). Alternation rates are depicted as percentages. Data are expressed as mean ± SEM (*n* = 10 per group). *^a^p* < 0.05 (first analysis for elastase and/or LPS effects). C, control; L, LPS; E, elastase; E + L, elastase plus LPS.

Using the T-maze spontaneous alternation test, the cognitive function was assessed 2 weeks after the last elastase exposure (T1) and at the end of the study (T2). At T1, a trend toward a lower alternation score in elastase-exposed mice was found (*p* = 0.060; Supplementary Figure 5). At T2, there was an overall significant difference among groups (Kruskal-Wallis: *p* < 0.05), representing a significant lower alternation score in elastase plus LPS-exposed mice compared to LPS-exposed mice (*p* < 0.05; Figure 8). In addition, there was a significant interaction effect between the model and the diet (*p* < 0.05). However, in the multiple comparisons test there were no significant differences between groups (Figure 8). No significant correlations were observed between alternation rates and serum cytokine levels (data not shown).

### Tight junction protein expression in brain microvessels

**FIGURE 9:**
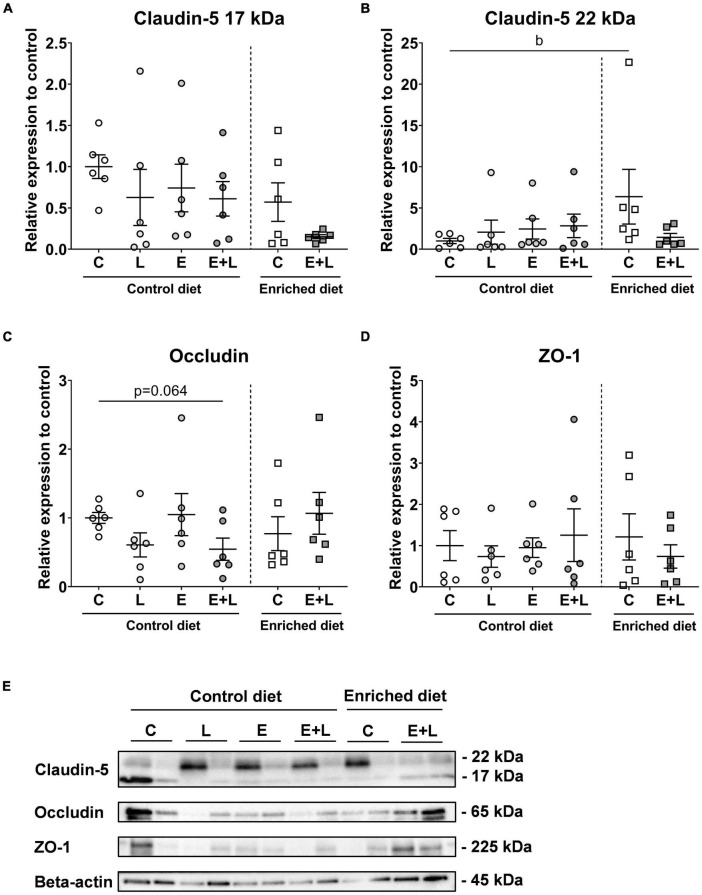
Tight junction expression levels in isolated brain microvessels after sacrifice at day 58. Expression levels of the 17 kDa **(A)** and 22 kDa **(B)**, claudin-5, occludin **(C)**, and ZO-1 **(D)** were quantified. Beta-actin normalized levels were compared to controls. Representative blots are presented in panel **(E)**. *^b^p* < 0.05 (second analysis for diet and/or E + L effects). Data are expressed as mean ± SEM (*n* = 6 per group). C, control; L, LPS; E, elastase; E + L, elastase plus LPS.

To measure the degree of BBB integrity, tight junction protein expression levels in isolated brain microvessels were compared. The expression levels of claudin-5 (both the truncated 17 and full-length 22 kDa forms), occludin and ZO-1 were measured. For the 17 and 22 kDa claudin-5, no significant effects of elastase and/or LPS were observed (Figures 9A,B). A significant effect of LPS was observed on occludin expression levels (*p* < 0.05), although multiple comparisons test did not reveal significant differences between groups (Figure 9C). No significant effects on ZO-1 expression levels were observed (Figure 9D).

For the 17 kDa claudin-5, although significant overall effects of both the model (*p* < 0.05) and the diet (*p* < 0.05) were observed, multiple comparisons test did not reveal any significant differences among groups (Figure 9A). In addition, a trend in interaction effect between the model and diet was found for the 22 kDa claudin-5 (*p* = 0.059; Figure 9B). For occludin, a significant interaction effect between the model and diet was observed (*p* < 0.05), and a trend toward lower expression levels was found in mice exposed to elastase plus LPS compared to control mice fed the control diet (*p* = 0.064; Figure 9C). This model effect was not observed in mice fed the enriched diet. For ZO-1, no significant effects were observed (Figure 9D). Representative bands are presented in Figure 9E. Tight junction expression levels did not show significant correlations with inflammatory marker levels in serum (data not shown).

### Microglial activation in the brain

**FIGURE 10:**
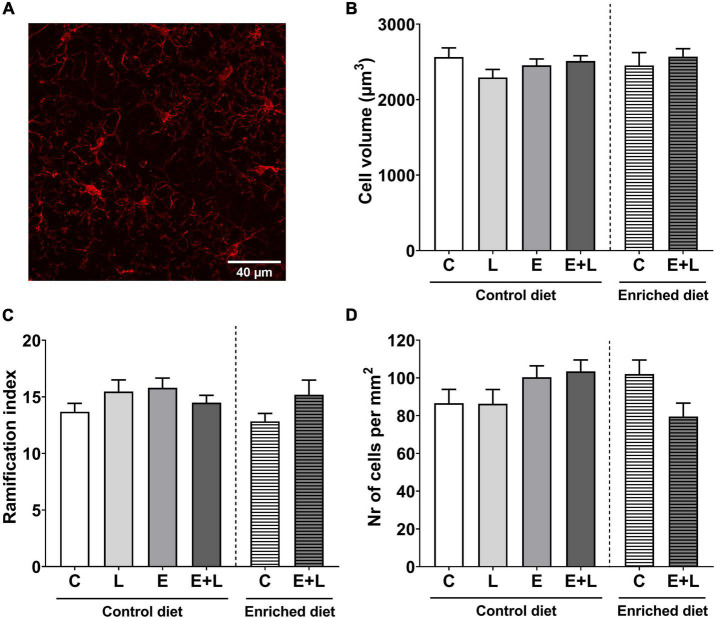
Microglia parameters in the CA1 region of the hippocampus. A 2D representation of a z-stack from the hippocampus in the control group stained with anti-iba1 (AlexaFluor 647) is presented in panel **(A)**. Total cell volume **(B)**, ramification index **(C)**, and the number of cells per mm^2^ **(D)** have been assessed. Data are presented as mean ± SEM (*n* = 6 per group). C, control; L, LPS; E, elastase; E + L, elastase plus LPS.

To assess the presence and degree of neuroinflammation in the brain, microglia cell volume, ramification index and numbers were determined in the ACC – an area of the PFC –, amygdala and hippocampus, which are brain areas involved in behavioral and cognitive functions. A representative 2D image of a z-stack in the hippocampus is presented in Figure 10A. In the hippocampus, no effects of elastase or LPS were observed on microglia cell volume or number of cells (Figures 10B,D). A trend in interaction effect between elastase and LPS on the ramification index in the hippocampus was observed (*p* = 0.077), although there were no significant differences between groups in the multiple comparisons test (Figure 10C). In addition, no effects on these parameters were found in the ACC (Supplementary Figures 6A–C) or amygdala (Supplementary Figures 6D–F).

In the hippocampus, no significant effects of the model nor diet were observed on microglia cell volume or ramification index (Figures 10B,C). Although for the number of microglial cells in the hippocampus the Kruskal-Wallis test showed a trend in difference between groups (*p* = 0.088), multiple comparisons did not reveal any significant differences (Figure 10D). In addition, no effects of the model nor diet on microglia parameters within the ACC or amygdala were observed (Supplementary Figure 6).

### mRNA levels of kynurenine pathway markers in brain

**FIGURE 11:**
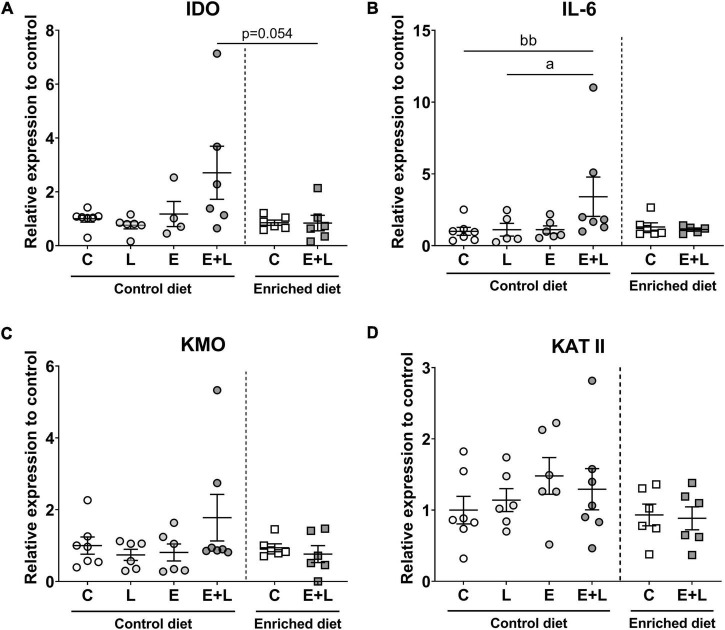
mRNA expression levels of kynurenine pathway markers in the PFC of the brain. These markers included IDO **(A)**, KMO **(B)**, IL-6 **(C)**, and KATII **(D)**. Relative expression levels to control are presented. Data are expressed as mean ± SEM. *^a^p* < 0.05 (first analysis for elastase and/or LPS effects). *^bb^p* < 0.01 (second analysis for diet and/or E + L effects). C, control; L, LPS; E, elastase; E + L, elastase plus LPS.

To assess whether the kynurenine pathway within the PFC is shifted, which may explain changes in behavior and may verify possible neuroinflammation, the mRNA levels of several markers of the kynurenine pathway – including indoleamine-2,3-dioxygenase (IDO), kynurenine aminotransferase II (KATII) and kynurenine 3-monooxygenase (KMO) – have been assessed within the PFC. In addition, IL-6 was included as one of the pro-inflammatory cytokines which can regulate the expression of IDO (66, 67). For IDO, KATII and KMO, no differences between groups were observed (Figures 11A,C,D). Elastase had a significant effect on IL-6 mRNA expression (*p* < 0.05). A higher IL-6 expression levels in elastase plus LPS-exposed mice as compared to LPS-exposed mice was observed (*p* < 0.05; Figure 11B). The ratio of KMO to KATII was calculated in order to assess whether a shift within the kynurenine pathway was present. A significant interaction effect between elastase and LPS was observed (*p* < 0.05), however, no differences between treatment groups were observed (Supplementary Figure 7).

There was a trend toward differences between groups for diet effects (*p* = 0.066; Kruskal-Wallis), and a trend toward lower IDO mRNA levels in elastase plus LPS-exposed mice fed the enriched diet compared to control diet fed elastase plus LPS-exposed mice was found (*p* = 0.054; Figure 11A). No effects of the model nor the diet were found for KATII or KMO mRNA levels (Figures 11C,D). There was a trend toward a model effect (*p* = 0.063) and a significant model × diet interaction effect (*p* < 0.05) for IL-6 mRNA levels. A higher expression of IL-6 was observed in mice exposed to elastase plus LPS compared to controls when fed the control diet (*p* < 0.01; Figure 11B). When comparing elastase plus LPS-exposed mice with control mice fed the enriched diet no significant difference on IL-6 mRNA levels was observed. No effects of the diet nor model were observed on KMO to KATII ratio (Supplementary Figure 7).

Overall, a trend toward a positive correlation was observed between IDO and KMO mRNA levels (*r* = 0.32; *p* = 0.064). No significant correlations between one of these markers with IL-6 mRNA was observed. In addition, KATII did not significantly correlate with IDO, KMO nor IL-6. When only including control diet fed animals, the trend toward a correlation between IDO and KMO mRNA was not observed anymore, although a significant correlation between IDO mRNA and IL-6 mRNA was observed (*r* = 0.61; *p* = 0.03). In contrast, when only animals fed the enriched diet were included, no significant correlations between any of the markers were observed.

Overall, no significant correlations between mRNA levels and cytokine levels in serum were observed (data not shown). Though, IL-6 mRNA levels significantly and negatively correlated with total distance walked in the open field test (*r* = −0.45; *p* < 0.01).

## Discussion

In this study, the impact of emphysema and pulmonary inflammatory events, as a model of COPD, on the brain and systemic outcomes has been evaluated. In addition, the therapeutic potential of a dietary intervention enriched with multiple components on emphysema, the degree of pulmonary, systemic and neuroinflammation and brain function in this elastase plus LPS-induced COPD model has been assessed.

With respect to the pulmonary phenotype, the model used in this study represents various characteristics of COPD pathology. First, elastase exposure resulted in significant emphysema, represented by an increased mean linear intercept value. No additional effects of LPS were observed on emphysema, which may indicate that elastase was the main driver of emphysema. No effects of the enriched diet were observed on lung emphysema. This can be explained by both the severity and irreversibility of the damage, and that alveolar wall destruction was induced before the start of the dietary intervention. Secondly, recurrent LPS exposures induced a significant inflammatory cell influx into the lungs, as represented by increased macrophage, neutrophil and lymphocyte numbers in BAL irrespective of elastase exposure. In addition to this, CRP and KC levels were increased, whereas VEGF-A was decreased in BALF, which was mainly caused by LPS exposure. CRP is involved in protective functions against for example bacteria (68) and is mainly produced in the liver, although it has also been suggested that it is produced locally in the lungs by alveolar macrophages (69). KC is the mouse equivalent of CXC motif chemokine ligand 8 (CXCL8) in humans, and is important for innate immune responses to triggers such as cigarette smoke, viruses and bacteria (70). In addition, the decrease in VEGF in the lungs might be an important contributor in the development of emphysema (71), and this change is mostly observed in the emphysematous dominant phenotype of COPD, whereas in the bronchitis dominant phenotype increases have been found (72). The present study shows that the effects of LPS on cytokine levels in the BAL fluid were less pronounced in elastase-exposed mice as opposed to the controls. In COPD, loss of innate immune responses are observed. This may, in part, be caused by exhaustion of responses from repeated insults (73, 74), but possibly also by reprogramming of alveolar macrophage responses to toll-like receptor 4 (TLR4) ligands, such as LPS, and the reduction of TLR4 expression in the respiratory epithelium (75–77). This failure of the immune system is suggested to be a very important component in the development and progression of COPD, and increases the susceptibility for infections and subsequent exacerbations (75). However, to be able to elucidate which processes might play a role, and to get more insight in the development of this immune dysfunction, assessment of immune activation and responses at multiple time points, i.e., after each subsequent LPS administration, and assessment of TLR4 expression is necessary. In addition, the mechanisms behind the increased CRP levels in the BALF of elastase plus LPS exposed mice fed the enriched diet are unclear, and further research is required to investigate the possible consequences for innate immune responses.

In contrast to the observed effects in the lungs, higher circulating levels of VEGF-A in serum were found, which are associated with the elastase exposure rather than the recurrent LPS exposures. This finding corresponds to previous observations in COPD patients where increased levels of VEGF in the circulation were found (78). Increased circulating concentrations of markers like CRP and CXCL8/IL-8 reflect systemic inflammation in COPD (78–80), especially as a result from acute exacerbations (78, 80). Although increased levels of CRP and KC in the circulation were observed in mice exposed to elastase plus LPS, these changes were only small and one reason for this might be the timing. Since mice were sacrificed at 10 days after the final LPS exposure, the inflammatory response might have partially restored. In addition, CRP levels in the lungs positively correlated with circulating levels of CRP, indicating that inflammation in the lungs might be related to systemic inflammation. Both CRP and KC are important for innate immune responses to triggers such as bacteria. In BALF, the additional effect of LPS on inflammatory markers was more pronounced than in serum. One explanation for this might be that the triggers have been administered in the lungs which causes a local inflammatory response. In the circulation, an inflammatory response might occur secondary and most likely in a lower rate as compared to the local responses within the lungs. Though, in the spleen, increased ratios of activated Th1 to Th2 cells in elastase plus LPS exposed mice were found, which was mainly the result of LPS exposure. The recurrent LPS exposures also resulted in increased numbers of activated Th1 cells in the spleen. Th1 cells are involved in adaptive immune responses to viruses or bacteria, and in inflammation and autoimmunity (81, 82). In accordance, increased splenocyte Th17 cell percentage was observed in response to LPS, which is also observed during bacterial responses and autoimmunity (81, 83). Interestingly, the percentage of Th17 cells was lower in emphysematous mice exposed to LPS as opposed to controls. Similar to the inflammatory cell and cytokines responses observed in the lungs of elastase plus LPS mice, these findings point to either exhaustion or insufficient immune responses to infectious triggers in emphysematous mice. Our findings also demonstrate that the enriched diet decreases the Th1/Th2 ratio, thereby improving the Th1 and Th2 cell balance in elastase plus LPS mice. This indicates that the enriched diet improves the derailed immune system function, possibly by improving gut health and immunity (84). However, no clear effects of the enriched diet on circulating CRP and KC were observed. Therefore, additional studies are necessary to further investigate the efficacy on inflammatory responses at multiple time points, such as during exacerbation (i.e., shortly after the LPS challenges). Potentially, combining the dietary intervention with commonly applied anti-inflammatory drugs in COPD, such as phosphodiesterase inhibitors, might be a useful future treatment strategy to target inflammation.

In COPD, various psychological and cognitive problems are common, including anxiety, depression and memory deficits. In this study, these brain-related impairments were present as a consequence of emphysema, with or without additional pulmonary inflammation. Firstly, pulmonary inflammation on top of emphysema was required to induce a significant decrease in locomotion. This reduced physical activity might reflect a reduction in exploratory behavior as a result of increased anxiety. In COPD patients, daily physical activity levels are frequently reduced (85), and mental health issues may contribute to this. Furthermore, the enriched diet increased the relative time spent in the inner zone to the time spent in corners of the open field in elastase plus LPS-exposed mice when comparing T2 to T1. This indicates that treatment with the enriched diet decreased anxiety-like behavior in the COPD model. Though, since no significant changes were observed in the COPD model fed the control diet, further research is necessary to assess the clinical relevance. Furthermore, it is likely that the open field test was not very sensitive for subtle changes in anxiety-like behavior. A test with a higher specificity for anxiety-like behavior, such as the elevated plus maze, might be more sensitive to subtle changes in behavior. Secondly, elastase exposure decreased cognitive function, i.e., spatial working memory, indicating that lung emphysema is related to impaired cognition. This corresponds to previous findings where cognitive function does deteriorate during exacerbations although may recover over time. In clinical studies, hypoxemia is an important factor in cognitive impairment in COPD patients, although it does not solely account for it (10, 86). The current study might confirm this since emphysema, together with airflow limitation, is the main driver of hypoxemia in COPD. Indeed, a single administration of 3 U elastase in mice resulted in both significant emphysema and decreased arterial oxygen saturation at 21 days post-exposure (87). Furthermore, an interaction effect between the model and the enriched diet was present, indicating that the dietary intervention improved cognitive function specifically in the mice exposed to elastase plus LPS. Although it did not reach significance, a higher alternation rate was observed in elastase plus LPS-exposed mice fed the enriched diet as opposed to control diet fed animals. Since the T-maze spontaneous alternation test is for assessing spatial working memory, the assessment of other cognitive domains with alternative tests might be useful to evaluate the therapeutic potential of the enriched diet on cognitive function more intensively. These domains include, but are not limited to, perception and memory, which are impaired in COPD patients (10). A relevant test might be the prepulse inhibition test, which is an assessment of sensorimotor gating (88).

The behavioral and cognitive functions that have been assessed in the current study are controlled by, amongst others, the brain areas ACC, amygdala, and hippocampus (89–92). As systemic inflammation is increased in the current model, one of the processes within the brain that might play a role is neuroinflammation, and one of the measures for this is microglial activation (93). This study shows that the activation of microglia cells is not changed in the COPD mouse model in any of the brain areas. Neuroinflammation can be induced and maintained by an impaired BBB (94). In this way, toxic compounds and proinflammatory cytokines can enter the brain in a higher degree and induce and maintain a neuroinflammatory state. This study showed that occludin expression in brain microvessels is reduced in elastase plus LPS-exposed mice, indicating reduced BBB integrity in the COPD model. Interestingly, the dietary intervention appeared to suppress these effects since this model effect was not present in the mice exposed to the enriched diet. Since the dietary intervention did not affect pulmonary nor systemic inflammation, it is likely that the compounds in the diet modulate BBB integrity through other mechanisms. It is not certain which exact mechanisms are involved although it is known that occludin, and its binding to ZO-1, can be affected by oxidative stress (95, 96). Although it is possible that the enriched dietary components regulate this directly or indirectly at the BBB, additional research is necessary to explore the involved mechanisms.

In the kynurenine pathway, tryptophan is degraded by IDO and, downstream in the pathway, by either KMO or KATs (97). Both in the periphery and the central nervous system, an increased activity of these enzymes can decrease the availability of tryptophan to be converted into serotonin. Serotonin is an important neurotransmitter regulating mood. Inflammation can induce depression by lowering tryptophan levels through, among others, the upregulation of IDO activity, and ultimately decrease serotonin production in the brain (98, 99). In addition, an inverse relation between kynurenine to tryptophan ratio and cognitive function has been observed in Alzheimer’s disease patients (100), highlighting a role of this pathway in cognition as well. In the brain, the kynurenine pathway can be shifted resulting in decreased production of the neuroprotective metabolite kynurenic acid and, through KMO, increased production of the neurotoxic metabolites 3-hydroxykynurenine and quinolinic acid (101). In the current study, elastase plus LPS-exposed mice showed overall increased mRNA levels of multiple markers of this pathway, i.e., IDO, which was related to KMO expression levels, together with a regulator of IDO and the pro-inflammatory cytokine IL-6 in the PFC. In addition, no changes in KATII expression levels were observed. This may indicate an imbalance of the kynurenine pathway within the brain, and thus an impaired serotonin metabolism and neurotoxic/neuroprotective metabolite shifts, in elastase plus LPS mice. An increased peripheral kynurenine to tryptophan ratio has indeed been observed in COPD patients and was related to the severity of the disease (47, 48). In addition, the intervention with the enriched diet seemed to decrease the expression of these markers in mice exposed to elastase plus LPS. It is likely that this is directly related to the tryptophan provided by the diet, although it may also be regulated through IL-6 since the increase in mRNA levels observed in elastase plus LPS exposed mice fed the control diet was not present in the enriched diet fed animals. Furthermore, KAT activity in the periphery may play a role since less KATs in the periphery may lead to higher circulating kynurenine levels and subsequently, more kynurenine may pass the BBB and impair brain function (102, 103). Therefore, it is possible that the intervention with the enriched diet resulted in more KATs in the periphery and lead to a more balanced conversion of kynurenine in the periphery and brain. However, our results are not conclusive as the sample size is low, there were no significant changes in KMO nor KMO to KATII ratio, and only mRNA levels were measured, which does not conclude the degree of activity or amount of the respective proteins. In addition, the activity and amount of protein are also dependent on the amount of substrate, which was not measured in the current study. Therefore, additional research is necessary to further examine whether the kynurenine pathway is changed in the COPD model by measuring levels of tryptophan, kynurenine and its neuroactive metabolites, and serotonin in the circulation and brain.

One of the major limitations of this study relating to the nutritional intervention is that the AIN-93M based control diet is rich in macro- and micronutrients preventing deficiencies. Whereas one of the major problems in COPD, and COPD-related comorbidities, is the nutritional deficiencies present in COPD patients (14, 15, 37–39). These deficiencies have not been modeled in this study and, therefore, this discrepancy with the situation in patients might have masked the potential of the enriched diet to intervene with the consequences of these deficiencies present in patients. Therefore, in future studies it would be valuable to include these nutritional deficient conditions. In addition, as mentioned before, hypoxemia and hypercapnia are important factors in COPD leading to cognitive impairment. However, in this study these pathological processes have not been examined. In order to know to what extent these processes were involved, this needs to be further elucidated in future studies. Furthermore, the gene expression analyses of kynurenine pathway markers have been limited to the PFC. It is valuable to measure the expression levels of these markers in the hippocampus as well, because of the cognitive impairments found in the current COPD mouse model and in COPD patients. This might provide insights into the involved mechanisms in the COPD-related cognitive impairment.

In conclusion, the presented COPD model shows both pulmonary and systemic inflammatory and brain-related impairments, including cognitive impairment, decreased locomotion as well as BBB integrity, and changes in kynurenine pathway markers within the brain. The intervention with the enriched diet applied in this study does not seem to have major effects on pulmonary or circulating inflammatory markers, although it did result in decreased Th1/Th2 ratios in this COPD mouse model. This study shows that the intervention with the enriched diet may have positive effects on anxiety and cognition, and improved the BBB integrity, and seemed to improve the balance of the kynurenine pathway in the brain. A nutritional intervention with a comparable composition is being evaluated in COPD patients (45), and therefore, the current study is highly relevant for the clinic. Overall, the presented mouse model does represent some brain-related symptoms which are observed in COPD patients, which is relevant for future studies relating to nutritional or pharmaceutic therapy efficacy assessment for limiting the impact of brain comorbidities in COPD.

## Data availability statement

The datasets presented in this article are not readily available because all authors must have insight into, and agree with, the goal and quality of the research performed with the dataset. In addition, data sharing should be done in a way that preserves intellectual property rights. Requests to access the datasets should be directed to CP, c.e.pelgrim@uu.nl.

## Ethics statement

This animal study was reviewed and approved by Central Committee for Animal Experiments (CCD), Den Haag, Netherlands.

## Author contributions

CP: investigation, methodology, formal analysis, project administration, writing—original draft, visualization, and data curation. IA, RB, AS-B, IF, TL-M, HJ, MD, and MK: investigation and validation. HG, RL, and RH: conceptualization, methodology, and resources. AS and JG: conceptualization and funding acquisition. SB: supervision, validation, and writing—review. GF, AH, and AK: conceptualization, funding acquisition, supervision, and writing—review. All authors contributed to the article and approved the submitted version.
