# Supplementary material for: Effects of a nutritional intervention on impaired behavior and cognitive function in an emphysematous murine model of COPD with endotoxin-induced lung inflammation
Source: Front Nutr. 2022 Nov 17;9:1010989. doi: 10.3389/fnut.2022.1010989 (PMC9714332; doi:10.3389/fnut.2022.1010989)
Supplement: Supplementary file 1 [file Data_Sheet_1.pdf]

Supplementary Material

Table S1. Composition of the control and enriched diet per kg of feed.

Component	Control diet (per kg feed)	Enriched diet (per kg feed)
**Digestible carbs**		
Cornstarch	445.7 g	449.4 g
Dextrinized cornstarch	148.3 g	149.6 g
Sucrose	100.0 g	100.0 g
**Fiber**		
Cellulose	47.5 g	13.3 g
Galacto oligosaccharides	0.0 g	27.2 g
Fructo oligosaccharides	0.0 g	2.2 g
Low viscosity pectin	0.0 g	4.8 g
**Protein & free amino acids**		
Casein	140.0 g	140.0 g
Tryptophan*	0.0 g	1.5 g
Arginine*	0.0 g	7.5 g
Aspartic acid*	0.0 g	3.8 g
Serine*	0.0 g	7.5 g
Cysteine*	0.0 g	1.5 g
Alanine*	26.6 g	0.0 g
**Fat**		
Soybean oil	40.0 g	27.6 g
Fish oil	0.0 g	12.4 g
- EPA	*0.0 g*	*3.5 g*
- DHA	*0.0 g*	*1.5 g*
**Others**		
Mineral mix AIN 93M**	35.0 g	35.0 g
Vitamin mix AIN 93M**	10.0 g	10.0 g
Vitamin D3	1000 IU	5000 IU
Vitamin B6 Pyridoxine	6.0 mg	12.0 mg
Vitamin B12	25.0 µg	75.0 µg
Folate	2.0 mg	6.0 mg
Vitamin C	0.0 mg	2000.0 mg

(Table S1 continued)

Vitamin E	75 IU	225 IU
Vitamin K	0.75 mg	7.50 mg
Nicotinamide	0.0 mg	120.0 mg
Zinc	30.0 mg	45.0 mg
Selenium	150.0 μg	330.0 μg
Choline Bitratrate	2.5 g	2.5 g
tert-Butylhydroquinone	0.008 g	0.008 g
Water	24.9 g	11.1 g
**Total nutrients**		
Protein	152.4 g	147.6 g
Carbohydrates	694.0 g	698.9 g
Fat	40.0 g	40.0 g
Dietary Fiber	47.5 g	47.5 g
Others	66.0 g	66.0 g
Energy	3865 kCal	3865 kCal

*added as free amino acid; **Premixes as described in Reeves et al.[1]

Table S2. Primer sequences used for qPCR of brain PFCs.

Gene	Forward primer (5' to 3')	Reverse primer (5' to 3')
Cyclophilin A	TTCCTCCTTTCACAGAATTATTCCA	CCGCCAGTGCCATTATGG
RPLP0	GGACCCGAGAAGACCTCCTT	GCACATCACTCAGAATTTCAATGG
Beta-2-Microglobulin	CTTTCTGGTGCTTGTCTCACTGA	GTATGTTCGGCTTCCCATTCTC
HPRT	TGGATATGCCCTTGACTATAATGAGTAC	AGGACTCCTCGTATTTGCAGATTC
IDO1	TGGAAAAGGCACTGCACGACA	TGGAAAAACGTGTCTGGGTCCA
IL-6	TGTGGACATTCCTCACTGTGGT	CCAACATTCATATTGTCAGTTCTTCG
KATII	TGATGGTTCAGCTCCTACCTCCT	TCAGTTGGGCCAATCTCTGG
KMO	AGCGTGGTGAGGAGTTTGTGT	CCCAAGCACGCAAACCGATG

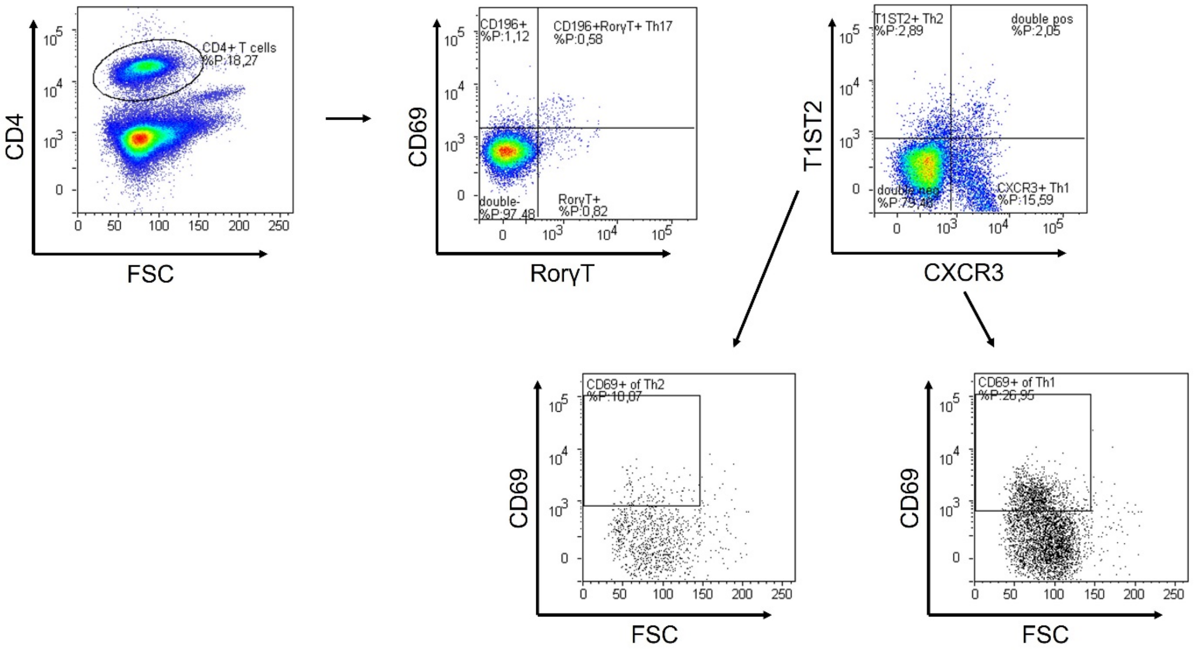

Figure S1. Flow cytometric analysis of T cell subpopulations in spleen. The gating strategy for selecting CD196+RorγT+ Th17 cells, CXCR3+CD69+ Th1 cells and T1ST2+CD69+ Th2 cells.

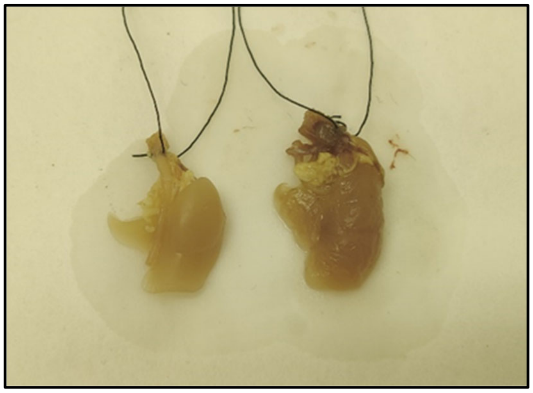

Figure S2. Macroscopic image of the right lobes of the lungs of a control animal (left) and an elastase-exposed animal (right).

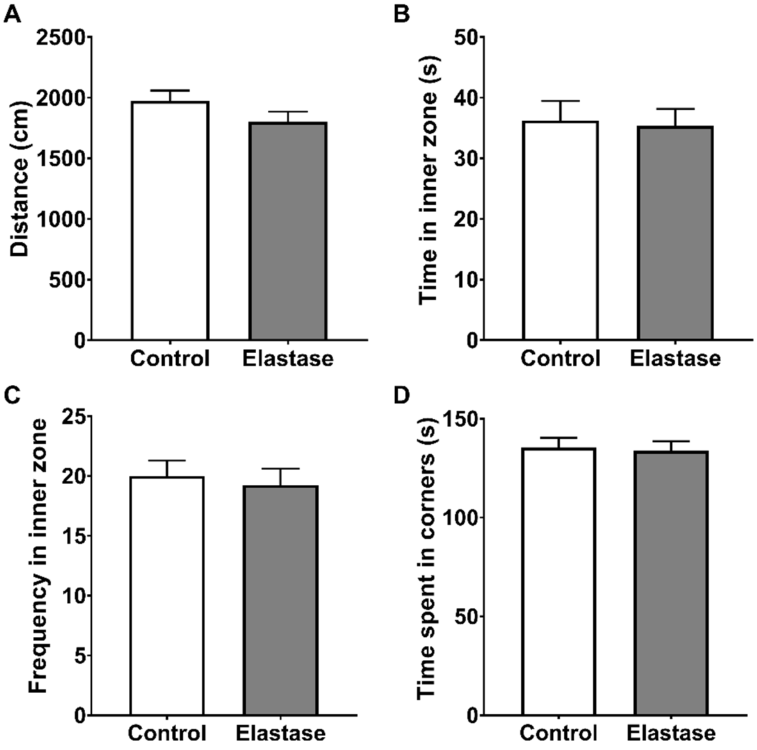

Figure S3. Open field behavior at day 25 in the study, after three elastase instillations and recovery (T1). Distance walked (**A**), time spent in the inner zone (**B**), frequency of entering the inner zone (**C**) and time spent in the corners (**D**) in the open field test. Data are presented as mean ± SEM (n=30 per group).

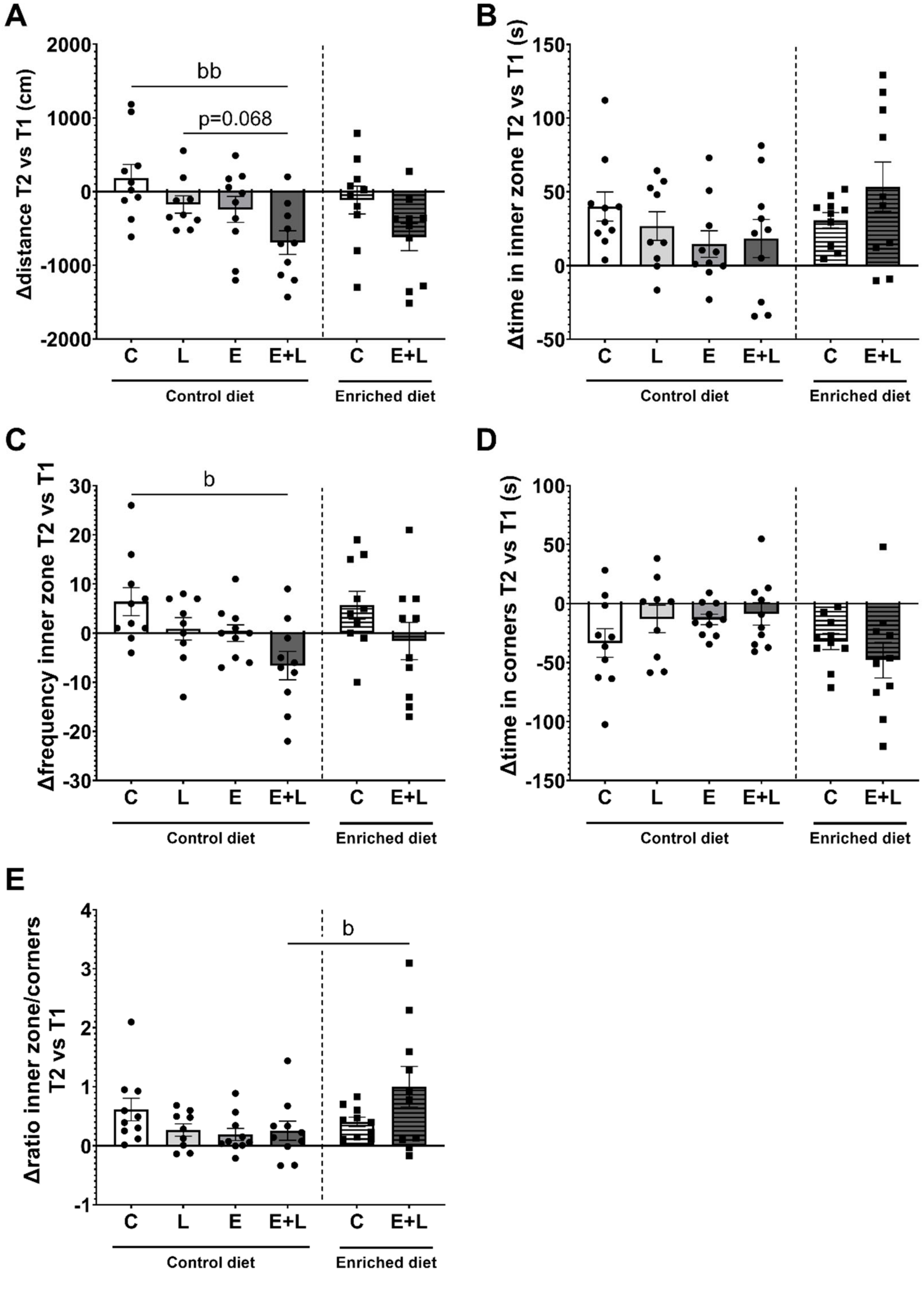

Figure S4. Differences in open field behavior on T2 versus T1. The results in distance walked (**A**), time spent in the inner zone (**B**), frequency of entering the inner zone (**C**), time spent in the corners (**D**) and the ratio time spent in the inner zone to time spent in the corners (**E**) of the open field. $^{b}p<0.05$; $^{bb}p<0.01$ (second analysis for diet and/or E+L effects). Data are presented as mean ± SEM. C: n=10 (control diet); L: n=9; E: n=10; E+L: n=10 (control diet); C: n=10 (enriched diet); E+L: n=10 (enriched diet).

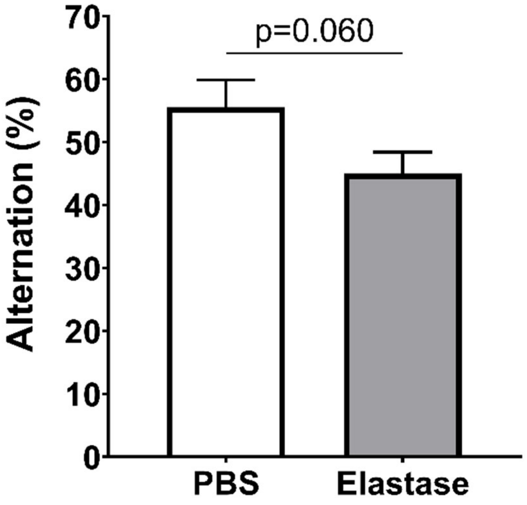

Figure S5. Cognitive function as assessed using the T-maze spontaneous alternation test at T1. Alternation rates are presented as percentages. Data are presented as mean ± SEM (n=30 mice per group).

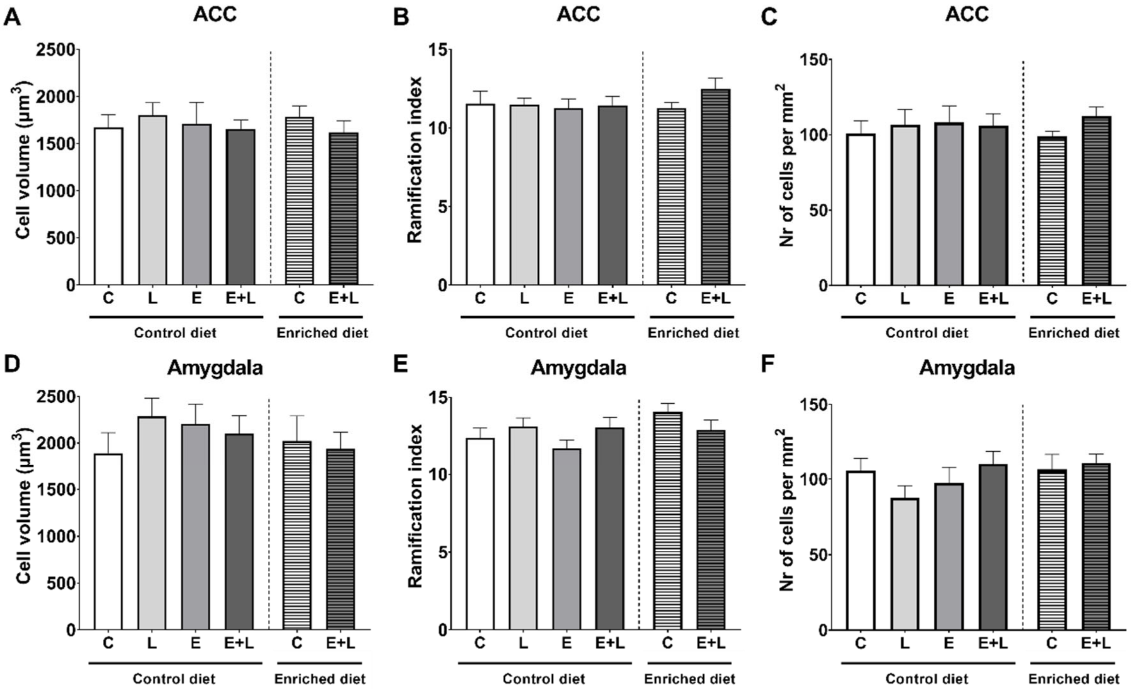

Figure S6. Microglial cell parameters in the ACC (**A-C**) and amygdala (**D-F**) within the brain. The total cell volume (**A, D**), ramification index (**B, E**) and number of cells per mm^2 (**C, F**) were determined. Data are presented as mean ± SEM. C=control (n=6); L=LPS (n=6); E=elastase (n=6); E+L=elastase plus LPS (control diet: n=6 (ACC), n=5 (amygdala); enriched diet: n=6).

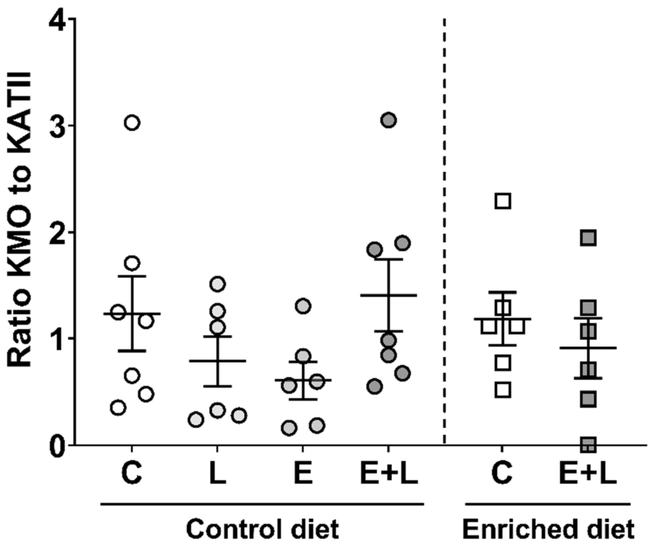

Figure S7. Ratio of KMO to KATII mRNA expression levels in the PFC of the brain. Data are expressed as mean ± SEM. C=control; L=LPS; E=elastase; E+L=elastase plus LPS.

References

1. Reeves PG, Nielsen FH and Fahey Jr GC. AIN-93 purified diets for laboratory rodents: final report of the American Institute of Nutrition ad hoc writing committee on the reformulation of the AIN-76A rodent diet. *The Journal of Nutrition* 1993; 123: 1939-1951. DOI: 10.1093/jn/123.11.1939.
